# Potential impact of diphacinone application strategies on secondary exposure risk in a common rodent pest: implications for management of California ground squirrels

**DOI:** 10.1007/s11356-021-13977-5

**Published:** 2021-04-21

**Authors:** Roger A. Baldwin, Theresa A. Becchetti, Ryan Meinerz, Niamh Quinn

**Affiliations:** 1 Department of Wildlife, Fish, and Conservation Biology, University of California, One Shields Avenue, Davis, CA 95616 USA; 2 University of California Cooperative Extension, 3800 Cornucopia Way, Ste A, Modesto, CA 95358 USA; 3 University of California Cooperative Extension, South Coast Research and Extension Center, 7601 Irvine Blvd, Irvine, CA 92618 USA

**Keywords:** Anticoagulant rodenticide, Diphacinone, Ground squirrel, *Otospermophilus beecheyi*, Rodenticide application, Secondary exposure

## Abstract

Anticoagulant rodenticides are a common tool used to manage rodents in agricultural systems, but they have received increased scrutiny given concerns about secondary exposure in non-target wildlife. Rodenticide application strategy is one factor that influences exposure risk. To understand the impact of application strategy, we tested residues of a first-generation anticoagulant (diphacinone) in liver tissue of radiotransmittered California ground squirrels (*Otospermophilus beecheyi*) following spot treatments, broadcast applications, and bait station applications in rangelands in central California during summer and autumn 2018–2019. We also documented the amount of bait applied, the mean time from bait application until death, and the proportion of ground squirrels that died belowground. We documented the greatest amount of bait applied via bait stations and the least by broadcast applications. We did not document a difference in diphacinone residues across any application strategy, although survivors had an order of magnitude lower concentration of diphacinone than mortalities, potentially lowering secondary exposure risk. We did not observe any difference among bait delivery methods in time from bait application to death, nor did we identify any impact of seasonality on any of the factors we tested. The vast majority of mortalities occurred belowground (82–91%), likely reducing secondary exposure. Secondary exposure could be further reduced by daily carcass searches. Results from this study better define risk associated with first-generation anticoagulant rodenticide applications, ultimately assisting in development of management programs that minimize non-target exposure.

## Introduction

Rodents cause extensive damage in many agricultural settings worldwide. One of the primary tools used to mitigate this damage has been anticoagulant rodenticides given the efficacy and cost effectiveness of this approach (Baldwin et al. 2014; Capizzi et al. 2014; Jacob and Buckle 2018; Witmer and Eisemann 2007). One of the primary drawbacks of anticoagulant rodenticides is the potential for secondary poisoning of predators and scavengers. Substantial effort has been undertaken over the last several decades to address secondary toxicity risks associated with anticoagulant rodenticides (van den Brink et al. 2018).

One proposed strategy for reducing secondary toxicity is to use application strategies that lower exposure risk to scavengers and predators (Buckle and Prescott 2018). Several researchers have postulated that the method by which rodenticide baits are applied could substantially affect anticoagulant residues in rodents (Dubock 1982; Record and Marsh 1988; Whisson and Salmon 2009). Commonly used strategies for rodenticide application in agricultural fields include spot treatments, broadcast applications, and bait stations (Jacob and Buckle 2018; Marsh 1994a, b; Salmon 2007; Tobin and Richmond 1993). Spot treatments involve the spreading of rodenticide baits over a label-specified area around a burrow entrance or rodent trail. This strategy is generally used over small areas given the time-consuming nature of this approach. Broadcast applications are used for treating larger areas. Broadcast applications involve the use of a spreader that is calibrated for distribution of bait over areas frequented by target species. Both spot treatment and broadcast applications take advantage of the natural foraging patterns of target rodent species (Marsh 1968; Matschke et al. 1983). Although poor seed foragers are generally not capable of feeding on enough bait to consume a lethal dose, these strategies may pose a risk to some non-target species. In areas where such non-target access is a concern, bait stations may be preferred. Many bait station designs exist, but a general premise is to eliminate non-target access to the rodenticide by animals that are larger than the opening of the bait station.

Many rodenticide labels now require the use of bait stations for rodenticide application given the potential reduction in primary non-target exposure. That said, bait stations could potentially increase secondary exposure by providing a constant bait supply that allows for repeated feedings at the station, as rodents do not reduce bait consumption until several days after initiating feeding (Whisson and Salmon 2002). This repeated feeding could ultimately allow for higher concentrations within the target rodent (Hindmarch and Elliott 2018). Conversely, broadcast applications have been postulated to have the lowest risk for secondary exposure given a sparse distribution of bait over target areas; enough bait is provided to allow the ground squirrel to consume a lethal dose, but perhaps not enough to allow for repeated feedings over several days. Spot treatments are believed to pose an intermediate risk when compared to the other two application strategies, as bait availability is lower than that for bait stations, but greater than that for broadcast applications given that target levels of bait from spot treatments are designed to allow for removal of multiple rodents per burrow system (Record and Marsh 1988; Salmon 2007). However, this assertion has not been rigorously tested. Knowing how differing rodenticide application strategies contribute to secondary toxicity risk would assist in the development of an application program that could reduce this risk.

An alternative strategy for reducing secondary exposure is to limit the amount of time between rodenticide consumption and mortality. Anticoagulant rodenticides require an extended timeframe for mortality to occur, often 4–13 days or more (Clark 1978; Hindmarch and Elliott 2018). As previously stated, animals can repeatedly feed on the bait during this timeframe, potentially leading to higher concentrations of rodenticides within the body than required to succumb to the toxicant (Hindmarch and Elliott 2018). Additionally, the longer an intoxicated rodent is alive and active on the landscape, the greater the opportunity for it to be predated upon (Buckle and Prescott 2018). This is of note given that several studies have suggested variable timeframes from bait application until death for target rodents depending on the application strategy (Baroch 1996; Whisson and Salmon 2009). A better understanding of how rodenticide application strategies influence time to death is needed to better guide applicators as to how to lower secondary toxicity risk to non-target predators and scavengers.

Ultimately, the best way to reduce secondary exposure is to eliminate intoxicated rodents from the food web (Record and Marsh 1988). This could be accomplished by eliminating the use of the anticoagulant by replacing it with a different toxicant that does not cause secondary exposure (e.g., zinc phosphide). However, anticoagulant rodenticides continue to be a key part of many rodent management programs given their high efficacy and cost effectiveness, ready availability and ease of application, reduced exposure risk when proper mitigation actions are taken, and a lack of effective and practical alternative management tools in many settings (Baldwin et al. 2014; Jacob and Buckle 2018). For these reasons, anticoagulant rodenticide use is likely to continue into at least the near future. Fortunately, many intoxicated rodents die within burrow systems, where they are unavailable to many predators and scavengers. However, the proportion that dies belowground is largely unknown. In a small study with California ground squirrels (*Otospermophilus beecheyi*), Whisson and Salmon (2009) determined that 3 of 8 (38%) radiotransmittered individuals died aboveground, while Saucy et al. (2001) reported similar results for water voles (*Arvicola terrestris*; 38% died aboveground). Knowing the proportion of the target species that die aboveground and remain available to scavengers is a key step toward devising management programs that reduce this secondary-exposure risk.

Discussions about secondary toxicity of anticoagulants have been increasing globally over the last decade (Quinn 2019; Rattner et al. 2014; Serieys et al. 2015), leading to numerous legislative attempts to limit or eliminate their use in many settings (Quinn et al. 2019). Even so, anticoagulant rodenticides are still considered an important tool for minimizing rodent damage in both agricultural and urban areas (Baldwin et al. 2014; Quinn et al. 2019). Ground squirrels (Sciuridae) provide an excellent example of this importance. Ground squirrels are broadly distributed in many regions of the world, and anticoagulant rodenticides are extensively used to mitigate damage caused by many ground squirrel species in North America (Askham 1994; Baldwin et al. 2014; Marsh 1994a). In California, the California ground squirrel is widely considered one of the two most damaging rodent species in agriculture (Baldwin et al. 2014; Marsh 1998). They are a common rodent species in the western USA, with a distribution that ranges from the state of Washington down to Mexico (Koprowski et al. 2016). Diphacinone is the most commonly used rodenticide for control of field rodents in California, and is available for use via spot treatments, broadcast applications, and bait stations depending on the particular product used (Timm et al. 2004). However, application strategy may influence non-target exposure risk, both by providing differing amounts of rodenticide bait, as well as by altering the foraging patterns of ground squirrels. Likewise, little is known about the availability of ground squirrel carcasses to predators and scavengers following anticoagulant baiting programs. Therefore, we established the following objectives to better elucidate potential risks associated with these factors. Specifically, we tested for (1) differences in amounts of diphacinone-treated oat groats applied via spot treatments, broadcast applications, and bait stations following current label requirements, (2) differences in time from application to death for each application strategy, (3) differences in concentrations of diphacinone residues in target ground squirrels for each application strategy, and (4) the proportion of ground squirrels that die belowground. Collectively, this information should allow for the development of management actions that can minimize non-target exposure associated with anticoagulant baiting programs.

## Materials and methods

### Study area

This study was centered on rangelands located on the western side of San Joaquin and Stanislaus Counties, CA, USA. These rangelands are seasonally grazed by cattle, with grazing typically occurring from October through March. The soils in the area consist of Carbona clay loam and Zacharias gravely clay loam with a small portion of Stomar clay loam up to an 8% slope. Annual precipitation in the area averages from 25.4–30.5 cm, with the majority occurring from October through March. Average temperatures range from a low of 4°C in January to a high of 35°C in July. Plant species composition was primarily annual grasses (non-native) and annual forbs such as *Hordeum murinum*, *Bromus madritensis*, *Bromus diandrus*, *Bromus hordeaceus*, *Avena fatua*, *Medicago polymorpha*, and *Erodium* spp. Forage production on Carbona clay soils can range from 2668–2825 kg ha^−1^ and 2522 kg ha^−1^ for the other soils in the area (Web Soil Survey [https://websoilsurvey.sc.egov.usda.gov/App/WebSoilSurvey.aspx]). Local forage production conducted on the ranch close to our study sites averaged forage production of 1636 kg ha^−1^ with a range of 479 to 2697 kg ha^−1^ (Becchetti et al. 2016; TA Becchetti, University of California, Division of Agriculture and Natural Resources, Modesto, CA; unpublished data). Small mammal species that were believed to be present at these study sites included desert cottontails (*Sylvilagus audubonii*), black-tailed jackrabbits (*Lepus californicus*), California voles (*Microtus californicus*), western harvest mice (*Reithrodontomys megalotis*), and deer mice (*Peromyscus* spp.).

### Plot establishment

During summer 2018, we established four 186 × 186 m plots (3.4 ha) in areas that had abundant ground squirrel numbers to allow for collaring with radiotransmitters. Plots were generally located at least 192 m ($\overline{x}$ minimum distance = 418 m) from any other plot to minimize the likelihood of a ground squirrel moving between plots, although the control and broadcast plots were separated by only 87 m during summer 2018. We believed that this distance would be sufficient to keep individual ground squirrels from moving between different treatment plots given that previous research identified a mean diameter of California ground squirrel home ranges of 20–34 m (Boellstorff and Owings 1995). This distance appeared sufficient, as we recorded only one ground squirrel out of 112 that was ever located within any treatment plot other than where they were captured. This individual was originally located in a bait station plot and showed up in a broadcast plot. However, the location within the broadcast plot did not occur until 7 days after the final broadcast application. Because bait rarely lasts 7 days in broadcast plots (Dochtermann 2005), it seemed unlikely that this ground squirrel ingested any appreciable amount of bait from the broadcast plot. As such, we considered all plots independent within each sampling season. Each plot was randomly assigned a treatment (spot, broadcast, or bait station) or served as the control. This design was replicated at new locations during summer 2019 and autumn 2018–2019, for a total of 16 plots, each 3.4 ha in size. All plots used in this study were similar with respect to vegetative cover, type, and aspect.

### Capture and collaring

To radiotransmitter ground squirrels, we established a trapping grid of 20–25 traps within the 0.4-ha core area of each plot. We selected this size to allow a buffer of 61 m on all sides of all plots to reduce the likelihood of ground squirrels moving off the treatment areas. This buffer distance is similar to other studies that have tested efficacy of rodenticides for California ground squirrel management (Baroch 1996; Salmon et al. 2007). We used Tomahawk wire cage traps (combination of 13 × 13 × 46 cm and 15 × 15 × 61 cm traps; Tomahawk Live Trap, Hazelhurst, WI) to capture ground squirrels. Traps were initially prebaited with plain oat groats for 1–2 days, and then were activated and again baited with oat groats to allow captures. Trapping occurred from early morning until 11:00 to reduce heat exposure to ground squirrels. Traps were checked approximately every hour. Upon capture, ground squirrels were moved within the traps to a shaded area for processing. We initially dusted ground squirrels with a 0.25% permethrin dust (Hi-Yield Garden, Pet & Livestock Dust, Voluntary Purchasing Groups, Inc., Bonham, TX) to reduce the potential for disease transmission from ectoparasites to field researchers. We handled ground squirrels via a cloth handling cone (Koprowski 2002). They were weighed, sexed, and fitted with a VHF transmitter via a cable tie around the neck (Model M1535, weight = 14 g; Advanced Telemetry Systems, Isanti, MN). The transmitters were retrofitted with a mortality signal that would trigger after 12 h of inactivity. No ground squirrels were collared that weighed <266 g to ensure that the transmitter did not constitute more than 5% of the ground squirrels body weight. Upon completion, we placed each ground squirrel back into the trap and released them at the site of capture. During summer and autumn 2018, we captured and collared 7 ground squirrels in each treatment and control plot (*n* = 28 in summer and *n* = 28 in autumn). We altered this strategy slightly in 2019 to collar 8 ground squirrels in each treatment plot and only 4 ground squirrels in control plots given the lack of mortalities that occurred in the control plots (*n* = 28 in summer and *n* = 28 in autumn). A total of 112 ground squirrels were collared for this study, with 30 each in bait station, spot treatment, and broadcast plots, and 22 in control plots.

### Radiotracking

We always allowed several days ($\overline{x}$ = 8.6 days, SE = 0.2, Range = 5–13) between collaring and the start of bait application to allow the ground squirrels a period of adjustment to wearing the collars. During this timeframe, we generally obtained locations of ground squirrels daily. Occasionally, other activities kept us from collecting locations before rodenticide application (we missed 2 days pre-treatment during summer 2018 and 1 day each during all other treatment periods), but we always identified ground squirrel locations daily following completion of initial rodenticide application until the termination of the trial. Locations were determined by walking up to ground squirrel locations using a 3-element Yagi antenna (Wildlife Materials, Inc., Murphysboro, IL), R-1000 receiver (Communications Specialists, Inc., Orange, CA), and known collar frequency. We documented if a ground squirrel was visually observed. Likewise, we documented any mortality that occurred, and any removed collars that were detected aboveground. In some situations, we could not easily find a ground squirrel location. If we could not find a location, we drove around for a minimum of 500 m beyond the buffer zone to continue searching for locations. If we could not detect a location, it was noted as missing for that day. Otherwise, we recorded all locations using a hand-held GPS unit.

### Bait application

For bait station plots, we used inverted “T”-shaped PVC pipe bait stations that are commonly used for California ground squirrel control (Whisson and Salmon 2009). The stations were made of 10-cm pipe with end caps cut in half and glued to the end of each bait station to keep bait from spilling onto the ground. The stations were 1.2 m in length and 0.9 m in height with an endcap on top to close off bait access. We spaced 64 bait stations 23 m apart following an 8 × 8 grid pattern that covered the entire treatment area (Baroch 1996), and we attached stations to metal T-posts that were staked into the ground. On day 0 for each bait application trial, we placed 0.9 kg of Rodent Bait Diphacinone Treated Grain (0.005%; California Department of Food and Agriculture, Sacramento, CA) into each bait station. We checked bait stations approximately every 3 days to maintain a bait supply within each station. If a station required refilling, we documented the amount of bait that was added. Bait station trials were conducted until bait was no longer removed from the stations (range = 14–19 days depending on season and year). Upon completion of the trial, we collected and weighed all bait from the bait stations. We subtracted this amount from the total amount applied to determine the total amount removed in each plot.

We also used the Rodent Bait Diphacinone Treated Grain (0.005%) for spot treatments. Before treatment, we identified all burrow openings within the 3.4-ha treatment area that appeared to house ground squirrels by looking for fresh footprints, scrapings, fecal pellets, and clear openings (i.e., devoid of detritus and spider webs and not overgrown with vegetation). We then treated each of these active burrow openings on day 0 of the trial with approximately 37 g of bait spread evenly over a 3.7–4.6 m^2^ area around the entrance of the burrow, making sure not to exceed 11.4 kg ha^−1^ per label requirement. However, if an individual treatment area overlapped multiple burrow openings, bait was applied only once over these openings to minimize rodenticide availability. This process was repeated 4 days later to ensure that ground squirrels had access to the bait over the period required to maximize efficacy (Whisson and Salmon 2002). We recorded the total amount of bait applied for comparison to other application strategies.

For broadcast applications, we initially used the Rodent Bait Diphacinone Treated Grain (0.005%) to allow for a more direct comparison of diphacinone residues within ground squirrels across the different application strategies. However, we did not observe any mortalities following application during summer 2018. As such, for the remaining 3 trial periods, we defaulted back to the label-specified rate of 0.01% diphacinone for broadcast applications (Rodent Bait Diphacinone Treated Grain [0.01%]; California Department of Food and Agriculture, Sacramento, CA). For broadcast applications, we first calibrated a seed spreader (Solo 421-S, Newport News, VA) to discharge the bait at the label-specified rate of approximately 11.4 kg swath ha^−1^. To calibrate the spreader, we used a combination of the swath width of distributed grain and the flow rate to approximate the desired application rate. Our seed spreader distributed bait at a width of approximately 2.9 m. This is less than that used in other studies (e.g., 9.1 m; Salmon et al. 2007), but more than that allowed on similar registered products (e.g., 1.9–2.1 m; PCQ, Bell Laboratories, Inc., Madison, WI; https://www.motomco.com/images/pdf/labels/780146-ca-pcq.pdf). We chose the seed spreader used in this study given that the resultant swath width represented an intermediate value between the two above-listed swath widths, while also allowing us to use a spreader that would be readily available to ranchers. We then flagged out transects that intersected active burrow systems throughout the treatment area to allow for efficient application. We applied bait along these transects on day 0 and again 4 days later to attain target exposure levels required for effective population reduction (Whisson and Salmon 2002). Trial periods for all three application strategies were operated concurrently for each season (summer 2018 = late June through mid-July, summer 2019 = mid-July through early August, autumn 2018 = early to mid-September, autumn 2019 = mid- to late September).

We followed label requirements for all bait application strategies, which was expected to result in differing amounts of bait applied via differing application strategies (e.g., Baroch 1996). Therefore, we compared the amount of bait applied across all three treatment types and two seasons using a two-factor ANOVA. If the model was significant, we used Fisher’s least significant difference *post hoc* test to determine which application strategies or seasons differed (Zar 1999).

### Fate of ground squirrels

We expected several outcomes of radiotransmittered ground squirrels including dropped collars, lost signals, unknown fates (collars that were recovered far from previous locations suggesting scavenging or for which we were unable to find a collar or ground squirrel when digging), squirrels that moved out of treatment areas before rodenticide applications occurred, unknown causes of mortality, rodenticide mortality, and survival. As such, we placed each radiotransmittered ground squirrel into one of these categories at the completion of each trial period, but for the purposes of this study, we censored all ground squirrels except those that survived within treatment or control plots or those that died from diphacinone exposure. We determined mortality rates by dividing the number of radiotransmittered ground squirrels that died from diphacinone exposure by the number of uncensored radiotransmittered individuals remaining at the end of the trial period.

For mortalities, if the ground squirrel carcass was aboveground, we dusted the ground squirrel with 0.25% permethrin dust, noted the location, and collected the animal. If the ground squirrel was belowground, we first pinpointed the location and then began digging. Soils were extremely hard and compact, requiring the use of a jackhammer to retrieve the ground squirrels. Depths of ground squirrels varied, but generally ranged from 0.5–1.2 m below ground. Once found, each ground squirrel was dusted with 0.25% permethrin dust, the condition of the carcass was noted, and the animal was stored in a freezer bag. We prioritized digging up ground squirrels the day the mortality signal was first heard, but initial staff limitations sometimes precluded us from digging up the ground squirrel until the next day. Upon liver collection, we noted that waiting an extra day often led to extensive decay, so adjustments were made to ensure carcass collection was on the day a mortality signal was first noted. This substantially improved our ability to retrieve intact and usable liver tissue. We also searched daily for any additional ground squirrel carcasses located aboveground in both treatment and control plots, and during digging activities, we collected any dead non-transmittered ground squirrels within burrow systems. We also searched for, and made note of, any non-target mortalities during our carcass searches. All collected ground squirrels were transported back to the laboratory where they were frozen for future laboratory assessment. We used Fisher’s exact test to test for seasonal differences in the proportion of ground squirrels that died belowground (Zar 1999).

At the end of each trial period, we again used Tomahawk wire cage traps to recapture surviving radiotransmittered ground squirrels using the same protocols outlined above, although we did not prebait during this process. All but two were recaptured (one in the control plot during summer 2018 and one in the broadcast plot during summer 2019). When we captured a radiotransmittered ground squirrel, we dusted it with 0.25% permethrin and then euthanized it via a carbon dioxide euthanasia chamber. All euthanized ground squirrels were collected and frozen for future liver extraction. Any non-transmittered ground squirrels that were captured were immediately released.

### Time to death

We estimated time from bait application to death by noting the day of initial application as day 0. Occasionally, a ground squirrel was retrieved aboveground without the collar emitting a mortality signal. Because the timeframe to initiate a mortality signal was 12 h, we considered those ground squirrels to have died the day they were recovered. For most ground squirrels that we collected that had emitted a mortality signal, we considered them to have died the day prior to initial detection given that all signal detections were completed before 12:00 each day. However, in some situations, the state of decay of the ground squirrel made it obvious that they had been dead for a longer period of time. We believe that surviving ground squirrels bumped or pulled on dead ground squirrels occasionally, keeping mortality switches from activating. In these situations, we made the assumption that the second day that the location did not change was the date of mortality, and we compared that estimated date of mortality to the condition of the carcass. For example, if initial instars of maggots were present, we considered the ground squirrel to have died 2–3 days prior. These two factors were corroborative in their estimation of when the ground squirrel likely died for all but one ground squirrel. For that ground squirrel, the carcass had completely decomposed, and as such, it was eliminated from further analysis. We used a two-factor ANOVA to test for potential differences in time from bait application to death and season. If the model was significant, we used Fisher’s least significant difference *post hoc* test to determine which application strategies or seasons differed (Zar 1999). All aspects of this project were approved by the University of California, Davis’ Institutional Animal Care and Use Committee (protocol no. 20025).

### Diphacinone residue analysis

We removed whole livers in the lab, froze them, and shipped them to the Texas A&M Veterinary Medical Diagnostic Laboratory in College Station, TX, for testing. We analyzed liver tissues for the presence of diphacinone using the quick, easy, cheap, effective, rugged, and safe method (QuEChERS) (Anastassiades et al. 2003). Following Vudathala et al. (2010), we prepared calibrators and controls in matrix (canine liver previously proven negative by this method). In every case, we weighed 1 g ± 0.10 g liver tissue into a 7-mL Omni bead vial pre-packed with 2.4-mm metal beads (OMNI p/n 19-670). All samples, positive and negative controls, and calibrators included an internal standard (ISTD = 100 μL of a 1 ng/μL diphacinone-d4 in acetonitrile [ACN] solution). We added approximately 0.2 g NaCl and 3 mL ACN to each vial, and closed all securely except to positive control for each run, to which we added 100 μL of a 1 ng/μL solution of diphacinone. We processed all calibrators, controls, and samples using an OMNI Bead Ruptor 12 Bead Mill Homogenizer (OMNI p/n 19-050A) for 1 min at 2.9 M/s, followed by 2 periods of 1 min each at 4.0 M/s. We added an additional 1-mL ACN to each vial, vortexed to mix, and centrifuged at speed sufficient to pellet all visible particles (~900×*g* for 5 min). We poured off the supernatant into QuEChERS tubes (pre-made in 15-mL conical polypropylene centrifuge tubes) containing 500-mg basic alumina, 250-mg C_18_ sorbent, 250-mg Florisil®, 175-mg MgSO_4_, and 50-mg primary-secondary amine. Tubes were vortexed thoroughly and centrifuged at a rate sufficient to collect packing (800×*g* for 5 min). We transferred the supernatant to 13 × 100 mm glass tubes, evaporated solutions just to dryness at 40 ± 5°C, and reconstituted each using 100-μL ACN. We transferred each extract to a 1.5-mL microcentrifuge tube and centrifuged at speed high enough to remove any flocculent material or remaining QuEChERS packing (6,500×*g* for 5 min). The supernatant was transferred to injection vials for analysis using an Agilent 6400 series LC-MS triple quadrupole mass spectrometer in negative ionization mode preceded by chromatographic separation over an Ascentis Express C18 column (2.1 mm × 100mm, 2.7 μm) with a mobile phase gradient consisting of 0.1% aqueous formic acid/0.1% formic acid in ACN (Bidney et al. 2015). Positive identification and quantitation were based on retention time, spectral matching, and verification of transition ions compared to a concurrently run certified reference standard.

We compared diphacinone residues using a one-factor ANOVA across five categories: spot treatment mortalities, broadcast application mortalities, bait station mortalities, survivors from rodenticide application plots, and control-plot survivors. If the model was significant, we used Fisher’s least significant difference *post hoc* test to determine which application strategies differed (Zar 1999). We also tested for differences in the concentration of diphacinone residues between 0.005 and 0.01% broadcast applications to determine if initial concentration of the rodenticide affected residue levels in ground squirrels (Student’s *t*-test; Zar 1999). Significance for all tests was set at α = 0.05. We used SAS 9.4 for all statistical analyses in this study.

## Results

The amount of bait applied varied substantially across application strategies (*F*_2,8_ = 290.5, *P* < 0.001), but did not vary across seasons (*F*_1,8_ = 0.6, *P* = 0.476). The greatest amount of bait was applied via bait stations ($\overline{x}$ = 64.1 kg, SE = 3.2), followed by spot treatments ($\overline{x}$ = 11.1 kg, SE = 0.4) and broadcast applications ($\overline{x}$ = 3.5 kg, SE = 0.1).

We censored a large number of ground squirrels in this study (*n* = 33), effectively lowering the number of ground squirrels used in analyses. Reasons for censoring included dropped collar = 13, lost signal = 9, unknown fate = 6, unknown cause of mortality = 3, and moved completely out of application site before application occurred = 2. We documented 46 radiotransmittered ground squirrel mortality events out of 57 remaining transmittered individuals within treatment plots, with 20, 19, and 7 occurring in bait station, spot treatment, and broadcast application plots, respectively (Table 1). This resulted in 100% mortality of transmittered ground squirrels for bait station and spot treatment applications, but only 39% mortality was observed for transmittered ground squirrels in the broadcast application plots. Of the mortalities in the broadcast plot, 100% occurred during autumn (Trials 2 and 4). We documented no mortalities in the control plot indicating that observed mortalities were due to diphacinone bait application (Table 1).

**Table 1:** The proportion (mortality) and associated efficacy values of bait station, spot treatment, and broadcast applications of diphacinone-coated oat bait (0.005% concentration unless otherwise noted) for radiotransmittered California ground squirrels (*n* = 30 for each)

	Control			Bait station			Spot treatment			Broadcast		
	Censored	Mortality	Efficacy	Censored	Mortality	Efficacy	Censored	Mortality	Efficacy	Censored	Mortality	Efficacy
Trial 1	0	0/7	0%	2	5/5	100%	2	5/5	100%	3	0/4	0%
Trial 2	0	0/7	0%	1	6/6	100%	5	2/2	100%	4	3/3	100%^a^
Trial 3	0	0/4	0%	2	6/6	100%	2	6/6	100%	1	0/7	0%^a^
Trial 4	0	0/4	0%	5	3/3	100%	2	6/6	100%	4	4/4	100%^a^
Comp	0	0/22	0%	10	20/20	100%	11	19/19	100%	12	7/18	39%

^a^These broadcast treatments were applied using 0.01% diphacinone-treated oats

Control plots with no bait application were provided for comparative purposes (*n* = 22). This study was replicated across four trial periods in rangelands located in central California during summer and autumn 2018–2019. Censored individuals were removed for a variety of reasons including a dropped collar, transmitter failure, unknown fate or causes of mortality, and ground squirrel movement out of the study area. Composite (Comp) data are provided for comparative purposes

We did not observe an impact of application strategy (*F*_2,41_ = 0.8, *P* = 0.462) or season (*F*_1,41_ = 1.5, *P* = 0.224) on mean time from bait application to death (collective $\overline{x}$ = 9.1 days, SE = 0.5, range = 4–19; Table 2). We did not detect a seasonal impact on the number of ground squirrels that died belowground (Fisher’s exact *P* = 0.336), with 91% of documented mortalities occurring belowground (Table 3). We observed five ground squirrels that we suspected were depredated or scavenged based on signs of feeding by predators or because the collar was found far from where the ground squirrel had ever been documented previously. If we considered those as aboveground mortalities that occurred from diphacinone exposure, then the adjusted belowground mortality rate drops to 82%. We still did not observe a seasonal effect following this scenario (Fisher’s exact *P* = 0.714).

**Table 2:** Mean, standard error (SE), and the range of the number of days from application of diphacinone-treated grain until death for California ground squirrels during summer and autumn in central California rangelands, 2018–2019

	Bait station				Spot				Broadcast^a^			
	Mean	SE	Range	*N*	Mean	SE	Range	*N*	Mean	SE	Range	*N*
Summer	8.5	1.1	5–16	11	8.9	0.6	6–12	11				
Autumn	10.0	1.8	4–19	9	10.3	1.5	4–17	7	8.1	0.7	5–11	7
Combined	9.2	1.0	4–19	20	9.4	0.7	4–17	18				

^a^No values are provided for broadcast applications during the summer season given that no mortalities occurred

Bait was applied via bait stations, spot treatments, and broadcast applications. Sample sizes (*N*) are provided for descriptive purposes

**Table 3:** Number of radiotransmittered California ground squirrel carcasses that were located belowground, aboveground, and the percentage located belowground at rangeland locations in central California during summer and autumn, 2018–2019

	Belowground	Aboveground	Percentage belowground	Potentially scavenged	Adjusted percentage
Summer	19	3	86	0	86
Autumn	23	1	96	5	79
Comp	42	4	91	5	82

We have also included information on the number of ground squirrels that were potentially scavenged (consumed or located far from previous locations) to represent the minimum percentage (adjusted percentage) that may have died belowground. Composite (Comp) data are provided for descriptive purposes

**Fig. 1:**
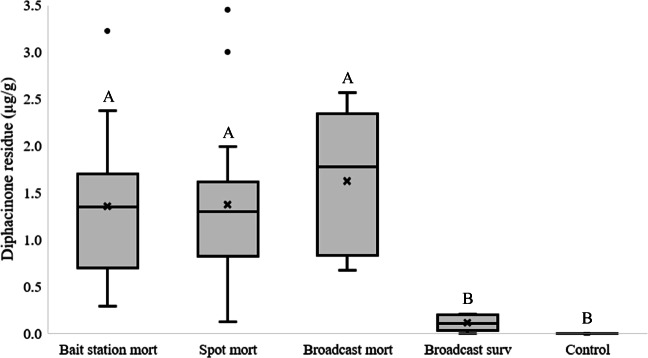
Box and whisker plot showing diphacinone residues (wet weight) in California ground squirrel livers following applications of grain bait via spot treatment, broadcast, and bait station application strategies in rangelands in central California. Most bait application strategies resulted in 100% mortality (mort), although some ground squirrels in the broadcast plots survived (surv). As such, residues from broadcast application mortalities and survivors were analyzed separately. Mean values are denoted by “x,” while median values are illustrated by lines in the middle of each box. Sample sizes for each category are the following: bait station mort = 18, spot mort = 15, broadcast mort = 5, broadcast surv = 10, and control = 17. Differences in mean values are denoted by different letters (*P* ≤ 0.05)

We observed a difference in diphacinone residues (wet weight) across the different treatments (*F*_4,60_ = 19.2, *P* < 0.001, *R*^2^ = 0.561), although this difference was driven by lower values observed in control plots and for ground squirrels that did not succumb to the toxicant (Fig. 1). We did not observe a difference in diphacinone residues across any of the three application strategies (Fig. 1), with an average residue of 1.399 μg/g (SE = 0.129) for mortalities across all three application strategies (*n* = 18, 15, and 5 for bait station, spot treatment, and broadcast mortalities, respectively). All ground squirrels that survived the diphacinone applications occurred in the broadcast plots (*n* = 10). We did not detect a difference in diphacinone residues of ground squirrels surviving broadcast applications of 0.005% ($\overline{x}$ = 0.085 μg/g, *n* = 4) and 0.01% ($\overline{x}$ = 0.130 μg/g, *n* = 6) diphacinone-treated grain (*t*_8_ = 0.9, *P* = 0.393). Collectively, residues for surviving individuals from diphacinone-treated areas averaged 0.112 μg/g (SE = 0.024). We observed no diphacinone residues in 14 of 17 ground squirrels in control plots, although three individuals exhibited very limited exposure (0.003, 0.003, and 0.01 μg/g).

## Discussion

Although bait stations are often considered a safer option for limiting primary non-target exposure to rodenticides, Whisson and Salmon (2009) speculated that bait stations might increase secondary exposure risk given an abundance of bait available for consumption by the target species ultimately allowing accumulation of toxicants in those animals. Similar to Baroch (1996), we did not observe this pattern with California ground squirrels in our study system, suggesting no increase in diphacinone residues when using bait stations for ground squirrel management. Ground squirrels did remove a substantial amount of grain from bait stations, exceeding the amount that was distributed by other application strategies. It is likely that much of that grain was stored underground for potential consumption later (Marsh 1994b; Whisson and Salmon 2009). The fate and risk of this stored grain remains unanswered. Baroch (1996) determined that diphacinone on 0.005% treated oats exposed aboveground degraded by 72% over a 9-day period, but only by 7% in bait stations. In a study on water voles (*Arvicola terrestris*), Sage et al. (2007) found that half-lives of bromadiolone baits in a simulated underground cache ranged from 24.6 to 42.7 days depending on the season assessed. However, bromadiolone has a substantially longer half-life than diphacinone which could influence the environmental deterioration time (Eason et al. 2008), and the Sage et al. (2007) study used wheat grains as a carrier. Rolled oat groats likely degrade more rapidly in the environment given the lack of a hard external coating, so degradation of diphacinone oat groats may occur more rapidly; at this point, it is unknown. At a minimum, caching behavior should be considered in locations where there is a concern of primary exposure to non-target burrowing rodents from these food stores (Whisson 1999; Whisson and Salmon 2009).

It is interesting to note that although less bait was applied via broadcast applications, diphacinone residues were as high as that observed in ground squirrels from spot treatments and bait station applications. This may have been driven at least in part by the higher concentration of diphacinone used in 3 of 4 broadcast applications, as other studies have noted a similar response in rodents when using higher-concentrations of anticoagulants (Kaukeinen 1982; Silberhorn et al. 2006; Ward 2003). However, the relationship between diphacinone concentration in treated grain and diphacinone residues in ground squirrel carcasses is not entirely clear. For example, Baroch (1996) noted no difference in concentrations of diphacinone from ground squirrel carcasses exposed to 0.005 and 0.01% diphacinone-treated grain. Likewise, we did not observe a significant difference in concentrations of diphacinone from ground squirrels that survived 0.005 and 0.01% applications, although we did note a general trend toward lower residue concentrations when using the lower bait concentration (0.01% $\overline{x}$ = 0.130 μg/g, 0.005% $\overline{x}$ = 0.085 μg/g). That said, it makes intuitive sense that lower concentrations of diphacinone bait should result in lower residues in target animals. Our initial goal was to use 0.005% diphacinone-treated grain for broadcast applications, but given a lack of mortality following our first application, we defaulted back to the label rate of 0.01% diphacinone-treated grain. In retrospect, this may not have been necessary, as the primary reason for the low efficacy observed in Trials 1 and 3 may have been due to low usage of the treatment areas by ground squirrels in these plots. For example, in the broadcast plots for Trials 1 and 3, ground squirrels were located within the treatment areas only 54% of the time (RA Baldwin, University of California, Davis, unpublished data). Conversely, ground squirrels were located within broadcast treatment areas 88% of the time for Trials 2 and 4 where 100% mortality occurred. Obviously, if ground squirrels do not have access to the bait, then a rodenticide application will be ineffective.

Likewise, the amount of bait available for consumption may have affected efficacy, as the swath width we used in our study was approximately 2.9 m compared to the 9.1-m width used by Salmon et al. (2007). This likely resulted in their treating 2–3 times as large of an area as us, and subsequently 2–3 times as much bait was likely applied. It bears noting that we observed a substantial difference in the efficacy of broadcast applications between the summer and autumn seasons. California ground squirrels tend to be less active during the heat of summer and sometimes will estivate for portions of the season (Marsh 1994b). This decreased summer activity, combined with less availability of grain from broadcast applications, could further explain the low efficacy observed for broadcast applications during summer. This all is of note, as previous research has indicated that 0.005% diphacinone applications were as efficacious as 0.01% concentrations in rangeland settings (Baroch 1996; Salmon et al. 2007). Although using a wider swath width might have increased the efficacy of broadcast applications in our study, it also may have resulted in higher residues of diphacinone in ground squirrel livers given a two- to threefold increase in bait availability. Using the 0.005% diphacinone bait may have lowered this risk, but given the increased amount of bait used when treating larger areas, combined with the levels of diphacinone residues observed in this study, it is unclear if and perhaps unlikely that these residues would have been lower than those observed for bait stations or spot treatments. Still, given the potential of lower residue concentrations and high levels of efficacy previously reported with 0.005% diphacinone broadcast applications, a switch to a lower-concentration product for broadcast applications in rangelands may be worthwhile. Further investigations should focus on the relationship between swath width of broadcast applications and concentrations of diphacinone-treated grain, both from an efficacy and a diphacinone-residue perspective, to better answer these questions. The impact of season on efficacy of broadcast applications should also be explored further.

Although we did not observe a difference across treatment types in diphacinone residues for ground squirrels dying from intoxication, we did notice a dramatically lower concentration in ground squirrels that survived. This suggests a substantially lower risk of exposure for non-target predators should they predate on a ground squirrel that was sublethally exposed. This risk would be further mitigated by the short half-life of diphacinone in ground squirrels (67–120 h in the liver; Ward 2003), indicating that long-term risk from sublethally exposed ground squirrels is substantially lower than that observed from scavenged ground squirrels or from predation on ground squirrels that had consumed a lethal dose but had not yet died.

Time from initial consumption to death can be a concern with anticoagulant rodenticides given the extended timeframe needed for them to work (Buckle and Prescott 2018; Record and Marsh 1988); the longer an intoxicated rodent is alive, the more opportunities exist for it to be predated. We did not note a difference in average time to death across any of the application strategies we tested, suggesting little impact of application type on this potential secondary exposure variable. Our investigation looked at the time from bait application to death, thereby accounting for the timeframe that it took for ground squirrels to first find and consume the toxicant, as well as how long it took for mortality to occur following ingestion. Therefore, we expected longer timeframes than those experienced in more controlled investigations, but results were consistent across studies (9–10 days; Clark 1978; Whisson and Salmon 2002). Previous investigations have suggested or observed increased time from application to ground squirrel population reduction across varying rodenticide application strategies, with bait stations generally taking longer given potential neophobic responses to bait stations, as well as territoriality of dominant males limiting conspecific access to bait stations (Baroch 1996; Whisson and Salmon 2009). Less availability of bait may have been a limiting factor in some previous studies, as Whisson and Salmon (2009) used much wider spacing of bait stations (39–92 m). This wider spacing likely led to exclusion of bait stations by dominant individuals, and perhaps took longer for ground squirrels to find and encounter bait stations. The tradeoff between cost of additional bait stations compared to quicker population reduction may be worthy of additional investigation. The cost of bait stations and rodenticide will certainly be much higher with shorter spacing, but a quicker time to death will reduce damage and will result in substantially less time required to perform daily carcass searches that are frequently required by many rodenticide labels.

Likely the best way to reduce anticoagulant exposure of predators and scavengers is to remove intoxicated individuals from the food chain. Most predators and scavengers of ground squirrels primarily hunt aboveground (e.g., raptors, coyotes, and bobcats). The vast majority of documented ground squirrel mortalities occurred within burrow systems (91%), effectively removing them from the food chain. We did note an additional five incidents where ground squirrels may have been scavenged or predated. If we assume that all these ground squirrels did or would succumb to diphacinone intoxication, this still resulted in >82% of the ground squirrels functionally unavailable to most predators and scavengers. The true proportion may have been somewhere in between. Regardless, the best strategy is to minimize the availability of carcasses to scavengers. Although they are time-consuming and challenging for land managers to implement, conducting daily carcass searches is important for removing dead ground squirrels from the food chain (Buckle and Prescott 2018; Montaz et al. 2014). Our daily searches resulted in the detection of only three non-transmittered ground squirrels that died aboveground. When combined with the four radiotransmittered ground squirrel carcasses located aboveground, we documented only 0.17 carcasses ha^−1^. Because of additional field activities and constraints on our time, we were only able to search for carcasses during mornings. Given that ground squirrels are diurnal while many predators and scavengers are nocturnal, carcass searches conducted shortly before nightfall might be most beneficial at removing these individuals from the landscape; the likelihood of ground squirrels dying aboveground at night seems low, but the potential advantage of conducting carcass searches in late afternoon has not yet been tested.

It bears noting that 33 out of 112 originally collared ground squirrels were censored due to a variety of reasons including dropped collars, lost signal, unknown fate, unknown cause of mortality, and moving completely out of the rodenticide application site. The impact of some of these factors could be reduced by taking a few proactive steps. The greatest loss was from dropped collars. This occurred from ground squirrels chewing off the collars or pulling them off with the collar intact. This potential could be lessened by ensuring a better fit. For example, we lost 5 of the 13 dropped collars during the first season. Learning how to fit the collars more snugly likely reduced some of these losses in subsequent seasons. We also lost 8 animals through signal loss, but none of these occurred during the first season. Because of budget limitations, we often reused some collars between study periods. For some transmitters, this may have led to battery failure. Therefore, using only new transmitters each season would likely reduce the number of animals lost through signal failure. Regardless, some loss should always be expected. Based on our results, we suggest planning to collar 20–30% more ground squirrels than the target goal for analyses to account for these potential losses.

## Conclusions

Many factors must be considered when determining how to manage rodent pests. How rodenticides are applied is an important consideration, as application strategies can influence the cost and practicality of each application strategy. For example, broadcast applications are generally considered the easiest and most economical strategy for ground squirrel management in large open areas (Kaukeinen 1982; Kowalski et al. 2006). Although we did not discern any difference in diphacinone residues in ground squirrels following any of the tested application strategies, we were unable to adequately test residues following a broadcast application of a lower concentration 0.005% diphacinone bait. Previous research suggests that a broadcast application of 0.005% diphacinone should be efficacious (Salmon et al. 2007), and may lower diphacinone residues, although collectively, this has not been rigorously examined. If effective, such a strategy would likely make broadcast applications the preferred rodenticide application approach for ground squirrel management in rangelands. At the time of this study, 0.01% diphacinone-treated grain products were registered for broadcast applications in California. Our study reflects the current application protocols for California ground squirrels in the state, and we observed no difference in residue concentrations across all three application strategies. Likewise, we did not identify any difference in time to death for any application strategy. Collectively, diphacinone residues and time to death suggest that spot treatments, broadcast applications, and bait stations have equivalent secondary-exposure risks based on the diphacinone concentrations included in this study. This risk is further mitigated by the fact that surviving ground squirrels had an order of magnitude lower diphacinone concentrations 2–3 weeks post-application, and the vast majority of ground squirrels died belowground, further reducing risk of secondary exposure. Ground squirrels also rapidly decayed belowground; if not recovered within 48 h, they were too deteriorated to use for diphacinone residue analysis and were covered in maggots within 72 h. This essentially eliminated fossorial scavenging unless it occurred within a few days post-mortality. Collectively, the low proportion of ground squirrels exposed aboveground, combined with daily carcass searches, should substantially reduce secondary exposure risk. It bears noting that rodenticide applications should be only one part of an integrated pest management program for rodent management (Baldwin et al. 2014; Hindmarch et al. 2018; Witmer 2018). Relying on anticoagulant rodenticides only when needed is the best strategy for minimizing the risk of secondary exposure.

## Data Availability

The datasets used and/or analyzed during the current study are available from the corresponding author on reasonable request.
